# Antiviral Therapy for Hepatitis C Virus: Beyond the Standard of Care

**DOI:** 10.3390/v2040826

**Published:** 2010-03-29

**Authors:** Leen Delang, Lotte Coelmont, Johan Neyts

**Affiliations:** Rega Institute for Medical Research, KULeuven, Minderbroedersstraat 10, 3000 Leuven, Belgium; E-Mails: leen.delang@rega.kuleuven.be (L.D.); lotte.coelmont@rega.kuleuven.be (L.C.)

**Keywords:** HCV, new antivirals, review

## Abstract

Hepatitis C virus (HCV) represents a major health burden, with an estimated 180 million chronically infected individuals worldwide. These patients are at increased risk of developing liver cirrhosis and hepatocellular carcinoma. Infection with HCV is the leading cause of liver transplantation in the Western world. Currently, the standard of care (SoC) consists of pegylated interferon alpha (pegIFN-α) and ribavirin (RBV). However this therapy has a limited efficacy and is associated with serious side effects. Therefore more tolerable, highly potent inhibitors of HCV replication are urgently needed. Both Specifically Targeted Antiviral Therapy for HCV (STAT-C) and inhibitors that are believed to interfere with the host-viral interaction are discussed.

## 1. Introduction

HCV is a positive sense single-stranded RNA virus which belongs to the family of Flaviviridae, genus Hepacivirus. The 9.6 kb HCV genome encodes for a large polyprotein, that, following maturation results in at least 10 proteins: the structural proteins C, E1, E2 and p7 and the non-structural proteins NS2, NS3, NS4A, NS4B, NS5A and NS5B [1]. HCV is a major cause of liver disease worldwide. An estimated 3% of the world population is infected with HCV, with 3 to 4 million newly infected individuals each year [2]. About 20% of patients with chronic HCV will ultimately develop cirrhosis and are at increased risk to develop liver failure and/or hepatocellular carcinoma. There is no vaccine available and such vaccine is also not soon expected. The current standard of care (SoC) for HCV infection consists of a combined therapy of pegylated interferon alpha (pegIFN-α) and ribavirin (RBV) administered for 24 or 48 weeks depending on the HCV genotype. The response rate to SoC varies according to virus titre, patient characteristics and genotype, with genotype 1 being the most difficult to treat (sustained virological response (SVR) of 40–50%) [3]. Furthermore the SoC therapy is associated with side effects such as flu-like symptoms, fatigue, depression, cognitive dysfunction, which has a serious impact on compliance [4]. Newer forms of IFN are in (clinical) development, from which albinterferon is the most advanced. Albinterferon consists of a fusion between IFN-α-2b and human albumin and has a significantly longer half-life than pegIFN-α. The results from the first large trials indicate that albinterferon plus RBV had an efficacy comparable to that of the SoC [5]. Taribavirin, previously known as viramidine, is a prodrug of RBV that is preferentially taken up by the liver. Unlike RBV, taribavirin is poorly taken up by red blood cells. In clinical trials taribavirin, when combined with pegIFN-α, resulted in a significantly lower rate of anemia, however the efficacy was lower compared to RBV-containing SoC [6].

Insights in the HCV life cycle and thus potential antiviral targets have long been hampered by the lack of efficient cell culture systems. However the generation of subgenomic HCV replicons, the HCV pseudoparticle model and more recently infectious HCV culture models were landmark developments that helped to the understanding of the life cycle of HCV and drug development [7].

## 2. Small Molecule Inhibitors of HCV Replication in Development

### 2.1. Virus-Specific Strategies

In theory, it should be possible to design selective inhibitors of every step in the replication cycle of HCV. The NS3/NS4A protease and the NS5B RNA-dependent RNA polymerase (RdRp) so far emerged as the most successful antiviral targets. Several inhibitors of the NS3 serine protease as well as nucleoside and non-nucleoside inhibitors of the NS5B polymerase are being or have been developed and some are currently in clinical trial. Inhibitors of other targets such as of the entry process, of the NS4A or NS4B protein and of the NS5A protein have only been recently identified.

#### 2.1.1. Entry Inhibitors

Attachment of HCV virions to the cell surface followed by internalization is the first step in a cascade of interactions between virus and host cell that is required for infection.

***ITX4520, ITX5061 (iTherx Pharmaceuticals).*** The small-molecule inhibitor ITX4520 interacts with SR-BI, a hepatocyte factor involved in the docking and entry of the virus [8]. Another small molecule inhibitor that was initially characterized as a p38 MAPK inhibitor, ITX5061, exhibits picomolar antiviral potency in HCV genotype 1 and 2 and is currently being evaluated in phase IIa clinical trials [9,10].

***Pro 206 (Progenics Pharmaceuticals).*** Pro 206 inhibits HCV entry at a post-attachment step and exhibits potent antiviral activity in the HCVpp model (EC_50_ = 2 nM) as well as in the HCVcc model (EC_50_ = 5.7 nM). Pro 206 and IFN-α-2a act *in vitro* (HCVcc) in an additive fashion. The development of Pro 206 has been stopped [11]. Second-generation lead compounds are in preclinical development [12].

***JTK-652 (Japan Tobacco).*** JTK-652 showed inhibitory activity against HCV genotype 1a and 1b pseudotyped viruses (in HepG2 cells and human primary hepatocytes). Multiple ascending doses of 400 mg (n = 9) and 800 mg (n = 9) JTK-652 TID or placebo for 14 days were safe and well tolerated in 8/12 healthy male subjects. However, in genotype 1 infected patients, there were no significant changes in HCV RNA to baseline after 29 days of 100 mg JTK-652 TID. Further development of this compound was halted [13].

***Cyanovirin-N***. The lectin cyanovirin-N (CV-N) was originally identified as an inhibitor of HIV-1 and HIV-2 replication [14], but exerts also *in vitro* anti-HCV activity. CV-N inhibits the infectivity of HCVpp and HCVcc at low nanomolar concentrations [15]. This antiviral effect results from an interaction of CV-N with N-linked glycans on the HCV envelope glycoproteins, thereby preventing the interaction of HCV glycoprotein E2 with the CD81-receptor.

#### 2.1.2. P7 Inhibitors

The HCV p7 protein is a “viroporin” (virus encoded ion channel) that is critical for HCV virus infection. P7 is critical for the release of infectious virions *in vitro* and *in vivo* [16,17]. When its ion channel activity is blocked, virus production is significantly reduced [18]. The sensitivity of this channel to small-molecule inhibitors renders p7 a promising target for novel therapies.

***BIT225 (Biotron Limited).*** BIT225, N-[5-(1methyl-1H-pyrazol-4-yl)-napthalene-2-carbonyl]-guanidine, was identified in a HCV p7 bacterial assay and was shown to be active *in vitro* against bovine viral diarrhoea (BVDV), a HCV related virus [19]. In a phase I study, safety and efficacy of two doses of BIT225 (35 and 200 mg, twice daily, 7 days) were examined in treatment-naïve or non-responder patients. The mean change in viral load was modest (with a maximum of −0.5 log_10_) in the 200 mg cohort [20].

#### 2.1.3. NS3 Protease Inhibitors

The amino-terminal domain of the multifunctional HCV NS3 protein forms, together with NS4A, a heterodimeric serine protease that cleaves the downstream region of the HCV polyprotein into four functional non-structural proteins [21]. The carboxy-terminal domain exerts a helicase/NTPase activity that unwinds the viral RNA [22]. Despite the fact that the substrate-binding cleft of the protease is rather shallow and largely hydrophobic, making it, in theory, a difficult target, several potent inhibitors are now in preclinical and clinical development.

***Ciluprevir (BILN 2061) (Boehringer-Ingelheim).*** The macrocyclic peptidomimetic BILN 2061 was the first HCV NS3/4A protease inhibitor to enter clinical trials [23]. When given to genotype 1 HCV patients during 2 days BID in monotherapy, BILN 2061 resulted in a rapid, dose-dependent decline of the viral load of 2–3 log_10_ IU/ml [24]. In genotype 2 and 3 HCV patients the effect was however less pronounced, indicating that treatment response may also depend on HCV genotype [25]. Drug resistance was rapidly selected *in vitro*, with a single amino acid mutation (either R155Q, A156T or D168V) in the NS3 protease being sufficient to confer resistance [26]. Because of cardiotoxicity in animal models the development of Ciluprevir was stopped.

***Telaprevir/VX-950 (Vertex Pharmaceuticals).*** VX-950 is a potent peptidomimetic (α-ketoamide) inhibitor of the HCV serine protease that was discovered using structure-based drug design [27] and is one of the most extensively studied STAT-C drugs. In contrast to BILN 2061, which is a non-covalent reversible inhibitor, VX-950 forms a covalent but reversible complex with the NS3/4A serine protease and has slow binding and dissociation kinetics [28]. Viruses (replicons) carrying the dominant resistance mutation for VX-950 *in vitro* (A156S) remain sensitive to BILN 2061, reciprocally the major BILN 2061-resistant mutants (D168V/A) are fully susceptible to VX-950 [29]. The variants A156T/V confer resistance to both BILN 2061 and VX-950 [30]. During a phase Ib clinical study, HCV genotype 1 patients were treated for 14 days with VX-950 in monotherapy at a dosage of 450 mg or 750 mg every 8 hours or 1250 mg every 12 hours (or placebo). A maximal median viral load reduction of 4.4 log_10_ IU/ml was reached in the optimal-dose group (patients who received 750 mg every 8 h) [31]. However viral breakthrough, associated with a number of mutations that confer low-level resistance (V36A/M, T54A, R155K/T, and A156S) and high-level resistance (A156V/T, 36 + 155, 36 + 156) to telaprevir, was noted in a significant number of patients during the second week of treatment [32]. The rapid selection of resistance in monotherapy, underlines the need for a combination therapy. Telaprevir was accordingly studied in combination with pegIFN-α in a group of treatment-naïve, genotype 1 HCV patients for 14 days. The effect on viral load was more pronounced with the combination therapy compared with either VX-950 or pegIFN-α monotherapy, with a mean decrease of 5.5 log_10_, 4.0 log_10_ and 1.0 log_10_ IU/ml respectively [33]. The safety of a triple therapy, in which VX-950 (750 mg every 8h) was combined with the SoC pegIFN-α-2a (180 μg/week) and RBV (1000 or 1200 mg/day), was consecutively studied in 12 treatment-naïve genotype 1 HCV patients for 28 days. All patients enrolled achieved undetectable HCV RNA levels by day 28. None of the patients experienced serious side effects [34]. These results warranted the set-up of 2 phase II clinical trials, named PROVE 1 and 2 studies, recruiting patients from the US and Europe, respectively. During the PROVE 1 study, treatment-naïve genotype 1 patients were randomized into 4 treatment groups, comprising standard therapy (pegIFN-α 180 μg/week and RBV 1000/1200 mg/day for 48 weeks); triple therapy *i.e.,* telaprevir (750 mg every 8 h) added to this SoC for 12 weeks, followed by an additional treatment period of pegIFN-α and RBV for 0, 12 or 36 weeks. The rate of SVR was significantly higher in the groups that received triple therapy compared to those who received the SoC (control group), 35% – 67% respectively, compared to 41%. Triple therapy resulted in the highest rates of rapid virological response (RVR or plasma HCV RNA < 10 IU/ml at week 4), *i.e.,* 81% *versus* 11% in the control group and low rates of relapse following cessation of treatment. Data were not substantially different between the groups receiving an additional standard therapy treatment for 12 or 36 weeks after triple therapy, indicating that a total treatment duration of 24 weeks (instead of 48 weeks) might be sufficient for the majority of patients [35]. The PROVE 2 study, conducted in Europe, had a similar study design as the PROVE 1 study, with as main difference that the group receiving triple therapy followed by standard therapy for 36 weeks, had now been replaced by a combination treatment of VX-950 and pegIFN-α (without RBV) for 12 weeks. The results from this study support the conclusions made for PROVE 1. The groups receiving triple therapy had greater SVR rates compared to the group receiving the SoC, *i.e.,* 60%–69% *versus* 48% respectively. In the group, receiving 12 weeks SoC following triple therapy, there was also a trend towards a higher SVR rate and a lower relapse rate than in the group in which patients received only triple therapy, favouring the addition of a period of pegIFN-α and RBV consolidation. The group treated with telaprevir combined with pegIFN-α without RBV had a low SVR rate (36%) and a high relapse rate (48%), highlighting the importance of RBV co-administration in future regimens [36]. In both trials, adverse effects such as rash, pruritis and anaemia were more common in the VX-950 containing treatment groups than in the groups receiving the SoC alone. Accordingly the discontinuation rates were higher for these groups compared to the control group. In the PROVE 3 trial, the efficacy of VX-950, in genotype 1 HCV patients who failed prior treatment with the SoC, was evaluated. During this phase IIb study telaprevir was combined with pegIFN-α with or without RBV. The enrolled patients can be categorized into non-responders, prior relapsers and prior breakthroughs to pegIFN and RBV treatment. The triple combination regimens (pegIFN/RBV/VX-950 for 12 or 24 weeks, followed by pegIFN/RBV for 12 and 24 weeks) resulted in significantly higher SVR rates (51% and 53% respectively), compared to the SoC regimen (14%). In more detail, 38% of prior non-responders, 76% of prior relapsers and 63% of prior breakthrough patients achieved a SVR. The group treated with telaprevir in combination with IFN without RBV had a SVR rate of 24%, again confirming the importance of RBV [37]. During two phase IIa studies (named study C209 and C210) telaprevir (750 mg every 8 h) was administered alone or in combination with the SoC in treatment-naïve genotype 2, 3 (study C209) and 4 (study C210) patients for 15 days. In genotype 2 patients telaprevir monotherapy, the combination with the SoC or the SoC alone resulted in a mean maximal reduction in viral load of −4.0 ± 0.49, −5.5 ± 0.24 and −4.0 ± 0.70 log_10_ IU/ml, respectively; while in genotype 3 patients the same strategies resulted in a reduction of −0.80 ± 0.33, −4.7 ± 0.32 and −4.5 ± 0.36 log_10_ IU/ml, for genotype 4 the changes in viral load were −1.4 ± 0.30, −3.50 ± 0.48 and −2.0 ± 0.40 log_10_ IU/ml, respectively. Thus telaprevir proved highly potent against HCV genotype 2, while its activity against genotype 3 was rather limited [38]. Telaprevir in combination with the SoC had a greater activity against HCV genotype 4 than the SoC or telaprevir monotherapy alone [39]. Telaprevir is currently being tested in phase III clinical trials, named ADVANCE, ILLUMINATE (in treatment-naïve patients) and REALIZE (in treatment-experienced patients).

***Boceprevir/SCH 503034 (Schering Plough/Merck).*** Boceprevir is another peptidomimetic (α-ketoamide) reversible covalent inhibitor of the NS3/4A protease, which showed potent *in vitro* antiviral activity and additive potency when combined with pegIFN-α [40]. Three mutations T54A, V170A and A156S conferred low to moderate levels of resistance to boceprevir, while A156T conferred the highest level of resistance *in vitro* [41]. During a phase Ib study, safety parameters and virologic response of the combination of SCH 503034 and pegIFN-α-2b were assessed in HCV genotype 1 non-responders. Patients received boceprevir (200 or 400 mg every 8h) as a monotherapy for 7 days, pegIFN-α-2b as a monotherapy for 14 days and the combination of both compounds for 14 days with “washout” periods between each treatment period. The combination treatment was well tolerated and resulted in an additive antiviral effect, with a mean maximal reduction in viral load of 2.88 ± 0.22 log_10_ IU/ml (pegIFN-α + 400 mg SCH 503034), while boceprevir monotherapy (400 mg) resulted in a reduction of 1.61 ± 0.21 log_10_ IU/ml and pegIFN-α-2b monotherapy in a reduction of 1.26 ± 0.20 log_10_ IU/ml [42]. Genotypic resistance analysis, in patients who received boceprevir (400 mg BID or TID) alone for 2 weeks, identified mutations V36M/A, T54A/S, V55A, R155K/T, A156S and V170A in the NS3 protease [43]. HCV genotype 1 treatment-naïve patients were enrolled in the SPRINT-1 phase II study, to evaluate the most effective treatment strategy. A higher dosage of boceprevir (800 mg TID) was evaluated in combination with the SoC in 5 different treatment regimens (*i.e.,* 4-week lead-in (or no lead-in) with SoC prior to the addition of boceprevir for an additional 24 or 44 weeks; triple therapy for 48 weeks with lower dose RBV) and compared to the SoC without boceprevir. The rationale for this lead-in treatment period with the SoC is based on the fact that both pegIFN and RBV reach steady-state concentrations by week 4. Patients thus receive the protease inhibitor first at a time when the backbone drug levels have been optimized, this may minimize the period of total treatment time required and may help reduce the likelihood for the development of resistance. SVR was significantly increased in the boceprevir arms compared to the SoC control arm. PegIFN and boceprevir combined with a low dose of RBV resulted in an increased viral breakthrough, relapse and lower efficacy, indicating the importance of RBV. Data suggest that a lead-in phase may be favoured in the patients treated for 48 weeks, but this needs to be further substantiated [44]. Only 18% of patients, who first achieved undetectable HCV-RNA after week 8, benefited from a longer treatment regimen of 48 weeks. Response-guided therapy based on week-8 viral response may thus offer a predictive tool to individualize therapy [45]. Safety data from the HCV SPRINT-1 study revealed that the most common adverse events reported in the boceprevir arms were fatigue, anemia, nausea and headache. The incidence of skin adverse events (rash or pruritis) observed in the boceprevir arms was comparable to that seen in the control arm. Boceprevir has also progressed to phase III clinical trials in which treatment-naïve (SPRINT-2) and prior non-responder (RESPOND-2) patients will receive a lead-in treatment with the SoC for 4 weeks prior to the addition of boceprevir.

***TMC435350 (Tibotec and Medivir AB).*** TMC435350 is a cyclopentane-containing NS3/4A protease inhibitor with potent *in vitro* anti-HCV activity and a high selectivity index (5.875), as demonstrated in a subgenomic 1b replicon system. The compound exerts synergistic activity in combination with IFN-α and a NS5B inhibitor, and additive activity when used in combination with RBV *in vitro*. It has a good tissue distribution and bioavailability [46]. Once daily TMC435350 given orally during a phase I study, was generally safe and well tolerated and demonstrated potent antiviral activity (median maximal reduction of 3.9 log_10_ IU/ml in viral load when given to patients at 200 mg once daily for 5 days) [47]. Currently TMC435350 is evaluated in a phase II clinical trial (OPERA 1), in which treatment-naïve and treatment-experienced HCV genotype 1 patients receive TMC435350 (25 mg daily, 75 mg daily, 200 mg daily, and 400 mg daily) or placebo with or without the SoC for 4 weeks, followed by SoC treatment until week 48 (or optionally, until week 24 for patients with an undetectable HCV viral load at week 4). Interim analysis of the cohort in which non-responders or relapsers to SoC received triple therapy with once daily TMC435350 (75–200 mg) or placebo for 4 weeks, revealed that triple therapy was well tolerated and resulted in potent antiviral activity with mean decreases in viral load of 4.3 – 5.3 log_10_ IU/ml, compared to 1.5 log_10_ IU/ml in the placebo group. The most common adverse effect was an influenza-like illness. Mild to moderate increases in bilirubin were observed in the highest doses group [48].

***ITMN-191/R-7227 (Intermune and Roche Pharmaceuticals).*** ITMN-191 has potent *in vitro* anti-HCV activity against NS3/4a proteases derived from genotypes 1–6. The combination of ITMN-191 with pegIFN resulted *in vitro* in a synergistic antiviral effect [49]. Furthermore the combination of ITMN-191 with the active moiety of either R1626 or R7128, which are both nucleoside analogue inhibitors of the HCV polymerase, resulted in enhanced antiviral activity and suppression of ITMN-191 resistant variants [50]. During a phase Ib study, genotype 1 treatment-naïve or prior non-responder HCV patients, were treated for 14 days with ITMN-191 BID or TID (total daily dose of up to 600 mg). ITMN-191 reduced HCV RNA in a dose-dependent manner with a maximal median decrease in HCV RNA of −3.8 log_10_ IU/ml in treatment-naïve and −2.5 log_10_ IU/ml in prior non-responder patients. No severe side effects were observed [51]. Viral variants (R155K) with reduced drug sensitivity were detected in a subset of patients that experienced a virologic rebound. When ITMN-191 (ascending doses ranging from 100 mg to 600 mg) was administered to treatment-naïve HCV genotype 1 patients in combination with the SoC for 14 days, this resulted in a pronounced decline in viral load of −4.7 to −5.7 log_10_ IU/ml, compared to −2.0 log_10_ IU/ml for the SoC. Combination therapy appeared generally safe and well tolerated [52].

***MK-7009/Vaniprevir (Merck).*** The macrocyclic non-covalent NS3-4A protease inhibitor MK-7009 proved to be a potent inhibitor (EC_50_-values in the nanomolar range) of *in vitro* anti-HCV replication. MK-7009 had reduced activity on the replication of replicons that carried the following mutations Q41R and F43S (3–5 fold), for R155K, A156T and D168Y (125–270 fold) [53]. During a phase IIa study HCV genotype 1 treatment-naïve patients received MK-7009 (ascending doses of either 300 mg BID, 600 mg BID, 600 mg QD, or 800 mg QD) or placebo in combination with pegIFN and RBV for 28 days. All patients continued pegIFN/RBV treatment for an additional 44 weeks. After 28 days of therapy, 69 to 82% of patients in the regimens containing MK-7009 *versus* 6% in the placebo group achieved undetectable HCV RNA levels. Interim results at week 12 indicated high rates of viral suppression to undetectable levels in patients treated with MK-7009 in combination with SoC for the initial 28 days (77–89% *versus* 60% in control (placebo) group). Resistant HCV variants at positions 155 and 168 were detected in viral breakthrough patients. No serious adverse events were observed during this study [54]. A phase IIb study has been initiated to evaluate the safety, tolerability and efficacy of MK-7009 when administered concomitantly with pegIFN/RBV to treatment-experienced patients with chronic HCV genotype 1.

***BI 201335 (Boehringer-Ingelheim).*** BI 201335 is a potent peptidomimetic HCV protease inhibitor. Ascending doses of BI 201335 were given to HCV genotype 1 treatment-naïve patients as a monotherpay for 14 days, followed by triple combination therapy with the SoC for an additional 14 days. BI 201335 monotherapy induced a pronounced virologic response with a median maximal reduction in viral load of −4.2 log_10_ IU/ml in the highest dose group (240 mg). However, a majority of patients in all dose groups experienced viral rebound during monotherapy. At higher doses an increased incidence of headache, gastrointestinal symptoms and unconjugated hyperbilirubinaemia was observed [55]. During a second study HCV genotype 1 treatment-experienced patients received ascending doses of BI 201335 (48 mg, 120 mg and 240 mg) in combination with the SoC for 28 days. In the highest dose group the median maximal reduction in viral load was −5.3 log_10_ IU/ml, also no viral breakthroughs were seen in this group, while this was observerd in 33% and 14% of patients in the 48 mg and 120 mg groups, respectively [56]. The predominant mutations in on-treatment viral rebound samples were R155K and D168V [57]. Safety and efficacy of BI 201335 was also assessed during a phase Ib trial, in which genotype 1 patients with compensated liver cirrhosis and non-response to previous SoC, were treated with 240 mg, once or twice daily, in combination with SoC for 28 days. All patients received a single loading dose of 480 mg of BI 201335 as the first dose. All patients showed a rapid and continuous decline in viral load with a mean reduction of −4.9 and −5.0 log_10_ IU/ml on day 28 in the 240 mg, once and twice dosing a day, respectively. No breakthrough was observed during treatment [58].

***Narlaprevir/SCH 900518 (Schering Plough/Merck).*** SCH 900518 has potent *in vitro* antiviral activity in the replicon system with EC_50_-values in the nanomolar range. The safety and antiviral activity were evaluated in a clinical study in which treatment-naïve and -experienced HCV genotype 1 patients received SCH 900518 for 7 days in monotherapy (at 800 mg TID or 400 mg BID + ritonavir). After a 4 week washout period the compound was administered together with pegIFN for 14 days, additionally patients received the SoC for 24 or 48 weeks. This treatment regimen resulted in a SVR in 81% and 38% of treatment-naïve and treatment-experienced patients, respectively. In a number of SCH 900518 dosed patients the variations R155K, A156T/S, V36M/L were detected [59 ,60]. During a phase IIa study (NEXT-1) treatment-naïve genotype 1 patients receive SCH 900518 (200 mg or 400 mg once daily) with ritonavir (100 mg daily) with the SoC for 12 weeks, with or without a 4 week lead-in period with the SoC, additionally they will be treated with 12 or 36 weeks of SoC. Interim results revealed that all treatment strategies resulted in a pronounced drop of HCV, with >85% RVR in the lead-in containing regimens [61].

#### 2.1.4. NS3 Helicase Inhibitors

The HCV helicase is believed to be essential for viral replication and forms a new attractive target for antiviral therapy. A few helicase inhibitors are in preclinical development; however none of them already passed to the clinical phase. Examples of molecules with reported anti-helicase activity are: benzimidazoles and benzotriazoles [62], the nucleotide-mimicking inhibitor QU663 [63], tropolone derivatives [64], an analogue of AICAR [65], acridone derivatives [66], a NS3 peptide [67], triphenylmethane derivatives [68], *etc*.

#### 2.1.5. Presumed Inhibitors of NS3/NS4A Interaction

NS4A is an essential cofactor to the NS3 protease that facilitates the membrane anchoring of the NS3-NS4A complex to the endoplasmatic reticulum (ER) and enables efficient polyprotein processing [69]. The interaction between NS3 and NS4A may be a good target for developing HCV inhibitors.

***ACH-806, ACH-1095 (Achillion Pharmaceuticals).*** ACH-806/GS9132 is an acylthiourea compound that binds selectively to NS4A, thereby preventing the formation of functional replication complexes. The reduced function of replication complexes in the presence of ACH-806 is not due to inhibition of NS5B polymerase nor NS3 serine protease. ACH-806 can thus be seen as an alternative protease inhibitor with a mechanism different of that of classical NS3/NS4A protease inhibitors. This unique mechanism may contribute to the lack of cross-resistance between ACH-806 and other NS3/NS4A protease inhibitors as was shown *in vitro* [70]. ACH-806 resistant replicons carry mutations (C16S and A39V) in the N-terminal region of NS3 which are in a region that interacts with NS4A. Intriguingly, no resistance mutations were observed in NS4A [71]. In a phase Ib trial in HCV genotype 1-infected individuals significant reductions in HCV viral load were observed (300 mg, BID; mean change in HCV RNA at day 5: −0.91 log_10_ copies/ml *vs*. +0.05 log_10_ for placebo) [72]. The development of ACH-806 was discontinued because of adverse effects. ACH-1095/GS9525 is the lead compound of the second generation of NS4A antagonists and has a similar mechanism of action. ACH-1095 has completed preclinical studies.

#### 2.1.6. Inhibitors of NS4B-HCV RNA Binding

HCV replication appears to be associated with intracellular membrane structures, the membranous web. This structure is believed to be induced by the NS4B protein. NS4B is also required to assemble the other viral non-structural proteins within the apparent sites of RNA replication. NS4B and HCV RNA have been shown to co-localize to the membranous web, suggesting that NS4B is in intimate contact with viral RNA in the context of authentic viral RNA replication [73]. Binding of the non-structural protein NS4B to the 3′-terminus of the HCV negative RNA strand is a recently identified target for drug intervention.

***Clemizole (Eiger Biopharmaceuticals).*** Clemizole hydrochloride, a first generation antihistamine, was found to have a substantial inhibitory effect on HCV RNA replication mediated by its suppression of NS4B’s RNA binding (EC_50_ for viral replication is ∼8 μM). Clemizole resistant variants carry mutations at position W55 and R214 in the NS4B protein [74]. A phase I proof-of-concept study, evaluating the safety and efficacy of clemizole as a single agent therapy in treatment-naïve patients infected with HCV genotypes 1 and 2, is underway.

#### 2.1.7. NS5A Inhibitors

The non-structural 5A (NS5A) protein has no intrinsic enzymatic activity, but likely exerts its functions through interactions with viral and cellular factors. It is a pleiotropic protein which plays an essential role in the HCV viral life cycle both by affecting the viral RNA replication as well as by modulating the physiology of the host cell to favor viral replication [75,76]. It occurs in a basally and hyperphosphorylated form, with different putative functions during HCV replication [77]. Furthermore NS5A, and more particularly the C-terminal domain III, would be the key factor for the assembly of infectious HCV particles [78]. NS5A consists of three domains, from which the structure of domain I has been resolved [79]. NS5A has become a promising new therapeutic target for the treatment of HCV.

***A-831 and A-689 (Arrow Therapeutics and AstraZeneca).*** A-831 (AZD2836) and A-689 (AZD7295), from which the structures have not yet been disclosed, have potent *in vitro* anti-HCV activity in the replicon system and good pharmacokinetic properties. A-831 and A-689 have different chemical structures and bind to different sites of the NS5A protein. Both compounds are in phase I clinical development [80]. Furthermore Arrow Therapeutics reported on a new class of compounds targeting the NS5A protein with improved potency. This series of compounds specifically reduced the hyperphosphorylated form of NS5A and induced NS5A redistribution in the cytoplasm [81]. The lead compound had nanomolar activity against different HCV genotype replicons, acted synergistically when combined with other direct HCV antivirals (STAT-C) or IFN-α and increased the genetic barrier of resistance when combined with other HCV inhibitors (against NS3 and NS5B). Several mutations located in domain I of NS5A conferred resistance to this lead compound. These replicon variants remained however sensitive to other checmically distinct (NS5A-targeting) compound series and were less fit than wild type replicon [82].

***BMS-790052 (Bristol-Meyers Squibb).*** BMS-790052 is a potent and specific HCV NS5A inhibitor, which demonstrated broad genotype activity *in vitro* with EC_50_-values ranging from the pM to the low nM range. It exhibited an additive to synergistic *in vitro* antiviral effect when combined with IFN-α and other small molecule HCV inhibitors. The compound most likely acts on the N-terminus of NS5A since drug resistance mutations mapped in this region [83,84]. The safety and efficacy of BMS-790052 was evaluated in a randomized, double-blind, placebo-controlled, single ascending dose (1, 10, 100 mg) study in HCV genotype 1 infected patients. All doses were well tolerated and had a safety profile similar to that of placebo. A mean maximal decrease in viral load of 1.8 log_10_ IU/ml and 3.3 log_10_ IU/ml after 24 h was achieved in patients, who received a 1 mg or 100 mg single oral dose, respectively [85]. BMS-790052 is currently being evaluated in phase II clinical trials.

#### 2.1.8. NS5B (RNA-Dependent RNA Polymerase) Inhibitors

The HCV NS5B protein is a RNA-dependent RNA polymerase (RdRp), which catalyzes the synthesis of a complementary minus-strand RNA, using the genome as a template, and the subsequent synthesis of genomic plus-strand RNA from this minus-strand RNA template. The HCV polymerase has the typical polymerase structure which is frequently compared with a right hand, where the palm domain contains the active site of the enzyme and where the fingers and the thumb are responsible for the interaction with the RNA. The fingertips are two loops that extend from the finger domain and that make contact with the thumb domain [86]. The HCV polymerase has been shown to be an excellent target for inhibition of HCV replication. Both nucleoside polymerase inhibitors and non-nucleoside polymerase inhibitors have been developed and several are or have been in clinical development.

##### a. Nucleoside polymerase inhibitors

Nucleoside analogues mimic natural polymerase substrates, causing chain termination and/or an increased error frequency when they are incorporated in a growing RNA chain. Generally, they show similar efficacy against all HCV genotypes, since the active site is well conserved among the distinct genotypes. All active site inhibitors of the HCV NS5B polymerase are ribonucleoside analogs.

***Valopicitabine (NM283) (Idenix Pharmaceuticals).*** Valopicitabine (NM283) is an oral prodrug of 2′-*C*-methylcytidine (NM107) [87]. 2′-*C*-methylcytidine was initially identified as a replication inhibitor of the bovine viral diarrhea virus (BVDV) and was later shown to inhibit HCV replication. Replicons resistant to 2′-*C*-methylcytidine carry the S282T mutation in the viral polymerase and show a reduced fitness compared with the wild-type replicon [88]. In clinical trials, valopicitabine was combined with pegIFN-α. After 48 weeks of treatment, the difference in decline of HCV RNA between the combination of pegIFN and valopicitabine and the combination of pegIFN and RBV was not significant [90]. Based on the overall risk-benefit profile observed in clinical trials, development of valopicitabine was stopped.

***R1626 (Roche and Pharmasset).*** R1626 is a prodrug of the nucleoside analogue 4′-azidocytidine (R1479) [91]. Replicons resistant to R1479 contain the S96T mutation or the combination of S96T and N142T in the viral polymerase. Interestingly, R1479 is not cross-resistant with valopicitabine [92]. The combination of R1626 with pegIFN or with pegIFN plus RBV was evaluated in a phase IIa clinical trial in patients infected with HCV genotype 1. A high rate of HCV infection relapse on combination therapy was observed [94]. Furthermore, 78% of patients treated with 3000 mg twice daily and 45% of patients treated with 1500 mg twice daily developed grade 4 neutropenia [95]. Because of fatal drug-induced lymphopenia, the development of R1626 was stopped at the end of 2008.

***R7128 (Roche and Pharmasset).*** R7128 is a diisobutyrate prodrug of PSI-6130, a cytidine nucleoside analogue (β-D-2′-deoxy-2′-fluoro-2′-*C*-methylcytidine) [96]. Replicons resistant to PSI-6130 contain the S282T mutation. However, this mutation results in a moderate three- to six-fold loss of sensitivity to PSI-6130. Thus, PSI-6130 presents a high barrier to resistance selection *in vitro*, selects for variants exhibiting only low-level resistance, and lacks cross-resistance with R1479 [97]. R7128 has shown antiviral efficacy in patients chronically infected with HCV genotype 1a and 1b (mean 2.7 log_10_ decline and maximum 4.2 log_10_ decline following 14 days of monotherapy of 1500 mg BID) [98]. Results of a phase I study of R7128 (500/1500 mg twice daily) in combination with pegIFN and RBV for 28 days demonstrated a significant antiviral effect (mean reduction in HCV RNA: 5.1 log_10_ IU/mL for 1500 mg R7128, 3.8 log_10_ for 500 mg, and 2.9 log_10_ for placebo) [99]. When HCV genotype 2 and 3 prior non-responders were treated with R7128 (1500 mg twice a day) in combination with pegIFN and RBV for 28 days, a mean viral load reduction of 5.0 log_10_ was observed and R7128 was generally well tolerated [100].

##### b. Nucleotide analogues

Nucleotide polymerase inhibitors are liver targeted prodrugs designed to enhance formation of its active triphosphate in the liver while minimizing systemic exposure of the nucleotide drug and its nucleoside metabolite. ***IDX-184 (Idenix Pharmaceuticals)***, a liver-targeted prodrug of 2′-methylguanosine monophosphate, demonstrated multilog viral load reductions in HCV-infected chimpanzees receiving 10 mg/kg for 4 days (mean HCV RNA decline: 1.4 to 3.8 log_10_ copies/ml) and appeared to be safe and well tolerated in healthy subjects at single doses up to 100 mg [101]. In treatment-naïve genotype 1 infected patients receiving 25, 50, 75 and 100 mg of IDX-184 once daily for three days and monitored for 14 additional days, the mean change in HCV RNA at the end of treatment was quite modest (−0.74 log_10_ for the 100 mg group) [102]. ***PSI-7851 (Pharmasset)*** is a prodrug of a uridine nucleotide analog PSI-6206 monophosphate. PSI-7851 demonstrated approximately 15- to 20-fold greater *in vitro* potency (EC_90_ = 0.31 μM) than PSI-6130 in a replicon assay [103]. Multiple oral doses of PSI-7851 (50 mg, 100 mg and 200 mg once daily for 3 days) were evaluated in 30 treatment-naïve HCV-infected patients. HCV RNA in the PSI-7851 treatment groups declined in a dose dependent manner after 3 days of monotherapy with mean reductions of −0.49, −0.61 and −1.01 log_10_ IU/mL for 50, 100 and 200 mg QD, respectively [104].

##### c. Non-nucleoside polymerase inhibitors

Non-nucleoside polymerase inhibitors are allosteric inhibitors that bind to less conserved sites outside the active site and inhibit the catalytic efficiency of the enzyme’s active site machinery by preventing a conformational transition needed for the initiation of RNA synthesis. Since the mechanism of action of non-nucleoside inhibitors differs from that of nucleoside inhibitors, cross-resistance between these two classes is unlikely to occur. A number of structurally unrelated series of non-nucleoside inhibitors have been reported; these include, but are not limited to, benzimidazoles, benzothiadiazines, thiophene derivates and benzofuranes. At least 4 different sites (A–D) have been shown to be targeted by non-nucleoside inhibitors; sites A and B are located in the thumb domain, sites C and D are located in the palm domain. In contrast to nucleoside inhibitors, a restricted spectrum of activity of non-nucleoside inhibitors against different HCV genotypes has been observed.

##### Thumb domain 1 (site A)

The first non-nucleoside polymerase inhibitor that entered clinical trials was ***JTK-003***. This benzimidazole compound is non-competitive with nucleotide incorporation and is believed to interact with an allosteric site on the surface of the thumb domain; drug resistant variants carry mutations at position P495 [107]. Inhibition of the viral polymerase results from the formation of intramolecular contacts between the thumb and the finger domain thus forcing the enzyme into an ‘open’, inactive conformation. JTK-003 was found to be well tolerated in phase I dose-ranging studies. The clinical development was however discontinued for unknown reasons.

Another class of non-nucleoside inhibitors that bind to RdRp site A, are indole-based inhibitors such as ***MK-3281*** and ***BILB-1941 (Merck, Boehringer Ingelheim).*** In preclinical studies, MK-3281 proved equipotent against HCV genotype 1a, 1b and 3a, but weak activity against genotypes 2a and 2b [108]. MK-3281 as monotherapy (800 mg MK-3281 BID for 7 days) was well tolerated and demonstrated strong antiviral activity against genotype 1b HCV with no evidence of viral breakthrough. Activity against genotype 1a and genotype 3 was however limited (mean maximum reductions from baseline of HCV viral RNA: 1.3 (0.15), 3.8 (0.19), and 1.2 (0.16) log_10_ IU/mL for genotype 1a/1b/3, respectively) [109]. Although BILB-1941 demonstrated reasonable activity in patients infected with HCV genotype 1 (viral load decreased by > or = 1 log_10_ IU/ml in 2/8, 2/8, 1/8, 2/7, 0/8, 2/8 and 4/5 patients on 60, 80, 100, 150, 200, 300 and 450 mg, respectively), the clinical development of this inhibitor was halted due to severe gastrointestinal intolerance [110].

##### Thumb domain 2 (site B)

***Thiophene carboxylic acid derivatives*** were reported to inhibit NS5B RdRp activity and HCV RNA replication in the replicon cell culture system [111]. Crystallographic studies revealed the existence of allosteric site B, a hydrophobic cavity located at the base of the thumb domain of NS5B [112]. Like site A inhibitors, thiophene carboxylic acid derivatives are non-competitive with nucleotide incorporation and inhibit an initiation step of RNA synthesis by interfering with conformational changes that are likely required for processive elongation of the replicating strand [113]. Thiophene-based inhibitors were found to select for Met^423^ en Leu^419^ resistant mutants in replicon cell culture experiment [114]. In 2006, a phase I proof-of-concept study was initiated with ***VCH-759 (Vertex).*** The drug was generally well tolerated and resulted in a significant reduction in viral load in treatment-naïve genotype 1 infected patients (> 1 log_10_ with a mean maximal decrease of 1.9, 2.3 and 2.5 log_10_ after dosing for 10 days at 400 mg TID, 800 mg BID and 800 mg TID) [115]. However, on-treatment rebound of viremia was observed, which suggested the rapid emergence of resistant strains. Indeed, genotypic analysis of NS5B variants of VCH-759 treated patients revealed mutations at positions 423 and 419 [116]. A second, more potent thiophene-based compound, ***VCH-916 (Vertex)***, has recently completed a phase I trial to assess the safety and efficacy [117]. Administration to healthy volunteers of doses between 50 and 600 mg did not result in any serious adverse effects. In genotype 1 HCV infected patients, VCH-916 achieved a mean maximal decline in HCV RNA of 1.5 log_10_ at doses of 200 mg TID and 300 and 400 mg BID over 3 days of monotherapy. Further development of this polymerase inhibitor in combination with other anti-HCV agents is being pursued [118].

In addition to thiophene-derived carboxylic acids, two other classes of molecules have been identified to target this allosteric site. ***Pyranoindole derivatives (Viropharma/Wyeth)*** (*i.e.*, ***HCV-371*** and a follow-up compound ***HCV-086***) entered clinical development but failed to demonstrate significant efficacy and development was therefore discontinued [119,120].

Another class of non-nucleoside polymerase inhibitors that interacts with site B is the class of the ***dihydropyranone derivatives*** [121]. ***PF-868554/Filibuvir (Pfizer)*** is a potent and selective HCV inhibitor *in vitro*. The predominant resistance mutation in resistant replicons is M423T [122]. A phase I clinical trial initiated with PF-868554 in 2006, revealed that the safety profile of this inhibitor (50/100/300 mg BID or 300 mg TID for 14 days) was similar to that observed in placebo. As monotherapy (100–450 mg BID or 300 mg TID for 8 days), PF-868554 demonstrated dose-dependent inhibition of viral replication, with mean maximum reductions in HCV RNA ranging from −0.97 to −2.13 log_10_ IU/mL [123]. In treatment-naïve genotype 1 infected patients triple therapy [PF-868554 (200, 300 or 500 mg BID) or placebo in combination with pegIFN (180 μg/wk) and RBV (1000/1200 mg/day) for 4 weeks] was associated with a mean reduction (log_10_ IU/mL) in HCV RNA of −2.10, −4.46, −4.67 and −3.62 at day 28 for placebo, 200, 300 and 500 mg BID respectively [124].

##### Palm domain 1 (site C)

Palm domain 1 is located at the junction of the palm and the thumb domains of NS5B and is in relatively close proximity to the catalytic site. The first class of palm domain 1 inhibitors was originally discovered by GlaxoSmithKline and is characterized by a ***benzothiadiazine*** scaffold. Akin to the thumb domain targeting compounds, benzothiadiazine-based compounds inhibit RNA synthesis before formation of an elongation complex [125]. *In vitro* benzothiadiazines select for Met^414^ resistant mutants [126]. ***ABT-333 (Abbott Laboratories)*** is another benzothiadiazine inhibitor that is currently in phase I clinical trials. In genotype 1 infected, treatment-naïve patients, treatment with ABT-333 + pegIFN/RBV resulted in significantly greater decreases in HCV RNA *versus* placebo + pegIFN/RBV (ABT-333 300 mg BID, 600 mg BID, 1200 mg QD, or placebo for 28 days (2 days monotherapy plus 26 days with pegIFN 180 μg/week + RBV 1000–1200 mg/d, weight-based)). The least square mean maximum HCV RNA change from baseline was −3.7, −4.0, and −3.5 log_10_ IU/mL for 300 mg BID, 600 mg BID, and 1200 mg QD ABT-333+pegIFN/RBV, respectively, compared to −1.4 log_10_ IU/mL for placebo+pegIFN/RBV [127]. The activity of yet another analog ***A-837093***, was studied in genotype 1a/1b HCV-infected chimpanzees (30 mg/kg BID for 14 days). Maximum viral load reductions of 1.4 and 2.5 log_10_ copies RNA/ml were observed for genotype 1a and 1b, respectively, within 2 days after the initiation of treatment, followed by rapid rebound due to the expected emergence of resistant virus variants [128]. ***Anadys Pharmaceuticals*** recently completed a phase Ib study with their benzothiadiazine-based inhibitor, ***ANA598***, which was well tolerated in healthy volunteers. ANA598 dosed 200 mg BID for 3 days as monotherapy resulted in antiviral activity, with median viral load decline of 2.4 log_10_ in treatment-naïve genotype 1 infected patients [129]. However, in a separate 14-day study with healthy volunteers conducted to extend the safety and pharmacokinetic profile of ANA598, a severe rash was observed in some ANA598 treated subjects. Three patients (two in the 800 mg once daily cohort and one in the 600 mg bid cohort) developed grade II rash and discontinued treatment after either six or seven days of consecutive dosing [130].

Another class of palm domain 1 binding compounds is the class of ***acylpyrrolidines (GlaxoSmithKline)*** [131]. ***GSK625433*** was advanced into phase I clinical trials but this study was halted because of adverse effects noted in long-term mouse carcinogenicity studies. [132].

##### Palm domain 2 (site D)

Palm domain 2 partially overlaps with palm domain 1, in proximity to the enzyme active site and the junction between the palm and thumb domains. A class of benzofurans ***(Viropharma/Wyeth)*** was identified as potent inhibitors of *in vitro* HCV replication [133]. These molecules select for Leu^314^, Cys^316^, Ile^363^, Ser^365^ and Met^414^ resistant mutants, which are almost all located in the allosteric palm domain 2 at the junction of the thumb and the palm domains of NS5B [134]. In phase I clinical trials, administration of 1000 mg BID ***HCV-796*** for 14 days in genotype 1 infected patients resulted in a mean maximal reduction in viral load of 1.4 log_10_ after 4 days [135]. In a combination trial of HCV-796 (doses ranging from 100 to 1000 mg BID) with pegIFN and RBV, mean viral load reductions at day 14 were 2.6–3.2 log_10_ for genotype 1 infected patients, increasing to 3.5–4.8 log_10_ in genotype non-1 infected patients [136]. Because of safety concerns, the development of HCV-797 was halted.

A class of ***imidazopyridines*** was identified as potent inhibitors of *in vitro* HCV replication [137]. Drug resistant variants carry mutations in the palm domain (C316Y) and inside the beta-hairpin of NS5B (C445F, Y448H, Y452H). This β-hairpin loop is located in close proximity to the catalytic active site and is believed to be involved in primer independent initiation of RNA replication. Within this class of imidazopyridines ***GS-9190 (Gilead)*** is currently being evaluated in phase II studies in combination with pegIFN and/or RBV.

### 2.2. Host-Specific Strategies

Cellular proteins that are essential for (efficient) HCV infection and replication may be targets for novel antiviral strategies. An advantage of HCV inhibitors that target host factors may be a higher genetic barrier than that of (most) inhibitors that target viral proteins. A disadvantage may be that side effects can be induced by inhibiting the primary roles of the host factors. We briefly discuss a selection of host factor-targeting inhibitors of HCV replication.

#### 2.2.1. Cyclophilin Binding Molecules

Cyclophilins are abundant proteins that are present in every organ. They display peptidyl-prolyl *cis-trans* isomerase activity which is important in *de novo* protein folding and isomerisation of native proteins [138]. There is a preponderance of evidence that cyclophilins play a crucial role in HCV replication. CypB has been proposed to be a functional regulator of the NS5B polymerase by enhancing its RNA binding activity [139]. Recently, CypA has been suggested as being the most indispensible cyclophilin for HCV replication [140–142]. Cyclosporine A (CsA), a widely used immunosuppressive drug, was shown to exert anti-HCV activity *in vitro* [143,144]. CsA binds to cyclophilins and inhibits their *cis*-*trans* peptidyl-prolyl isomerase activity. The compound forms an inhibitory complex with cyclophilins that blocks the phosphatase activity of calcineurin in activated T-cells. Tacrolimus (FK506), an immunosuppressive drug that interacts with calcineurin but not with cyclophilins, exerts no anti-HCV activity indicating that the immunosuppressive activity is not a prerequisite for potency against HCV [145]. A number of non-immunosuppressive cyclophilin binding compounds are currently in (clinical) development.

***Debio 025/Alisporivir (Debiopharm).*** Debio 025 is at least 10-fold more potent than CsA as a HCV-inhibitor, with an EC_50_-value in the nanomolar range (in the subgenomic replicon and infectious HCV system) [146]. The *in vitro* combination of Debio 025 with either SoC or STAT-C inhibitors [NS3 protease or NS5B (nucleoside and non-nucleoside) polymerase inhibitors] resulted in an additive antiviral activity. Debio 025 had the unique ability to clear hepatoma cells from their HCV replicon when used alone (at low concentrations) or in combination with IFN and/or STAT-C inhibitors. At concentrations that are readily achieved in human plasma (0.1 or 0.5 μM), Debio 025 was able to delay or prevent the development of resistance to HCV protease inhibitors as well as to nucleoside and non-nucleoside polymerase inhibitors *in vitro* during a combined colony formation assay. *In vitro* selection of Debio 025 resistant replicon proved to be a very lengthy process. The compound remained active against replicons that were resistant to various NS3 protease- and NS5B polymerase inhibitors. Conversely, these STAT-C inhibitors retained wild type-activity in Debio 025 resistant replicon cultures [147]. Mutations identified in Debio 025 resistant replicons reside mainly in domain II of the NS5A gene (Con1 subgenomic replicon) [148] or near the cleavage site between NS5A and NS5B (JFH1 subgenomic repicon) [149].

In phase I clinical studies HCV/HIV-coinfected patients received Debio 025 in monotherapy (1200 mg BID), a mean maximal decrease in viral load of 3.6 log10 IU/ml was observed after 15 days [150]. The efficacy and safety of Debio 025 was further evaluated in a phase II study in which HCV genotype 1, 2, 3 and 4 treatment-naïve patients were randomized to receive escalating doses of Debio 025 (200, 600, 1000 mg/day) combined with pegIFN-α-2a or monotherapy with either 1000 mg/day Debio 025 or 180 μg/week pegIFN-α-2a for 4 weeks. In patients with genotype 1 and 4, the 600 mg- and 1000 mg - combination treatment resulted in a −4.61 ± 1.88 and −4.75 ± 2.19 log_10_ IU/ml decrease in viral load at week 4, respectively. Reduction in viral load in genotype 2 and 3 infected patients was more pronounced with reduction up to −5.91 ± 1.11 and −5.89 ± 0.43 log_10_ IU/ml, respectively, with the same treatment regimens [151]. The 200 and 600 mg dose were very well tolerated, whereas the 1000 mg dose was associated with isolated and reversible hyperbilirubinemia linked to inhibition of biliary canalicular transporters. However bilirubin levels returned to baseline after treatment cessation. Subsequently the effect of treatment with Debio 025 (400 mg or 800 mg) in combination with pegIFN-α-2a and RBV was studied in HCV genotype 1 patients that were non-responders to the SoC. Debio 025 at doses of 400 mg (with initial loading dose) and 800 mg daily for 29 days resulted in a significant decrease of HCV viral load of −1.96 – 2.38 log_10_ IU/ml, respectively, when combined with the SoC [152].

***NIM811 (Novartis).*** NIM811 is another oral non-immunosuppressive cyclophilin binding inhibitor, which exhibits also stronger *in vitro* anti-HCV activity than CsA. The combination of NIM811 with a protease inhibitor and (non)-nucleoside polymerase inhibitors resulted in an additive to synergistic effect, as tested *in vitro.* As is the case for Debio 025, it is much more difficult to develop resistance against NIM811 than against viral specific inhibitors. Furthermore no cross-resistance could be observed between these inhibitors, and NIM811 prevented, like Debio 025, the emergence of resistance development against STAT-C inhibitors [153]. When NIM811 at a dose of 600 mg B was given in combination with pegIFN-α-2a to genotype 1 HCV infected patients that previously failed on SoC, a mean drop in HCV RNA of −2.78 log_10_ IU/ml was observed after 14 days of treatment, compared to −0.58 log_10_ IU/ml decrease in the IFN monotherapy arm. The major safety concern was a decrease in platelets and an increase in serum bilirubin [154].

***SCY-635 (Scynexis).*** SCY-635 is also a non-immunosuppressive analogue of CsA that exerts potent anti-HCV activity *in vitro*. SCY-635 inhibited the peptidyl-prolyl *cis-trans* isomerase activity of CypA at nanomolar concentrations. The combination of SCY-635 with RBV or IFN-α-2b resulted in an additive to synergistic effect, as tested *in vitro* [155]. During a phase Ib clinical study HCV genotype 1 patients were enrolled into one of three ascending cohorts (total daily dose: 300, 600, 900 mg) for 15 days. No serious adverse events were observed. Consistent decreases in plasma RNA were observed only in the 900 mg cohort. Group mean and median nadir values were 2.20 and 1.82 log_10_ below baseline [156].

#### 2.2.2. Glycosylation Inhibitors

*N*-glycosylation of proteins is important for optimal folding and assembly into complexes. Proper folding and assembly of many viral glycoproteins is assisted by ER-resident chaperones [157]. Some chaperones interact with glycoproteins by binding to their *N*-glycan moieties. Before this interaction can take place, the *N*-glycans need to be modified by the trimming of two terminal glucose residues. This process is catalyzed by two ER-resident enzymes, α-glucosidases I and II [158]. In the lumen of the ER, HCV glycoproteins E1 and E2 are modified by *N*-linked glycosylation on well conserved sites among HCV genotypes [159]. Inhibition of the activity of ER-α-glucosidases results in misfolding/misassembly of the HCV glycoproteins and thereby leads to an accelerated degradation of E1-E2 heterodimers and a decrease in availability of these complexes for viral budding and morphogenesis. However, glucosidase inhibitors can cause side effects *in vivo* because of off-target inhibition of various glucosidases. Since HCV is particularly sensitive to ER-α-glucosidase inhibition compared with host proteins, glucosidase inhibitors should be specific for ER-α-glucosidases to avoid side-effects.

***Iminosugars.*** Iminosugar derivatives of the glucose analog deoxynojirimycin (DNJ) were identified as α-glucosidase inhibitors and exert broad antiviral activity including against HIV, influenza virus, flaviviruses, pestiviruses (BVDV), hepadnaviruses (HBV & WHV) and the hepatitis C virus (HCV) [160]. Inhibition of *in vitro* HCV replication by DNJ and analogues has been shown to be related to an effect on morphogenesis and a reduction of infectivity of virions that are residually released under treatment [18,161]. Recently, the anti-HCV activity of two alpha-1-C-alkyl-1-DNJ derivatives was reported. These molecules have different selectivity patterns towards gastro-intestinal oxidases compared to previously studied DNJ derivatives, but their anti-HCV activity was found to be slightly lower than the anti-HCV activity of the parental molecules, due to lower inhibition of ER-glucosidases [162].

***Celgosivir (Migenix).*** Derivatives of the naturally occurring castanospermine, a well-known inhibitor of ER-α-glucosidase I, are reported to inhibit *in vitro* anti-BVDV activity [163]. The antiviral efficacy and safety of one of these derivatives, 6-O-butanoyl-castanospermine (Celgosivir/MX-3253), were demonstrated before in clinical trials in HIV-1-infected patients. Celgosivir used as monotherapy (200 mg once/twice daily, 400 mg once daily) was well tolerated in all dosage groups and resulted in a modest antiviral effect in treatment-naïve or IFN-intolerant genotype 1 HCV infected patients. Results of a phase II study in genotype 1 HCV-infected non-responder or partial responder patients showed that triple therapy of celgosivir (400 mg once daily), pegIFN and RBV produced a substantial decrease in viral load compared to current standard therapy (mean decrease of HCV RNA −1.63 log_10_IU/ml *vs*. −0.92 log_10_IU/ml for SoC therapy) [164]. Clinical development of celgosivir is currently put on hold.

#### 2.2.3. Statins

HCV requires elements of the cholesterol and fatty-acid biosynthetic pathways for efficient replication [165]. Lipoprotein receptors (LDL receptor, SR-BI receptor) have been reported to be involved in HCV entry [166]. Nevertheless, the role of the LDL-receptor in HCV entry is still uncertain. Cholesterol metabolism is also required for the assembly of VLDL particles. These lipoprotein particles have been shown to complex with HCV virions in serum [167]. Cholesterol- and sphingolipid-lowering compounds, like statins and myriocin, have been reported to inhibit HCV replication [168].

***Statins.*** Statins, which are widely used for the treatment of hypercholesterolemia, inhibit 3-hydroxy-3-methyl-glutaryl coenzyme A (HMG-CoA) reductase, the rate-limiting enzyme in cholesterol biosynthesis in the liver. HMG-CoA reductase catalyzes the conversion of HMG-CoA to mevalonic acid [169]. Besides its cholesterol-lowering effect, statins were shown to inhibit the replication of subgenomic HCV-1b replicons and to suppress RNA replication of JFH-1 HCV [170,171]. *In vitro* combination studies of statins with IFN and selective HCV inhibitors (protease inhibitor VX-950, nucleoside polymerase inhibitor R1479, non-nucleoside inhibitors HCV-796 and GSK-4) resulted in an additive antiviral activity. Importantly, mevastatin delays or even prevents the development of HCV-796 escape mutants. Hence, the combination of selective HCV inhibitors with statins may also have the potential to delay or even to prevent resistance development in the clinical setting [172]. The precise mechanism of the anti-HCV activity of statins has not yet been unraveled. The anti-HCV activity of statins may possibly result from inhibition of geranylgeranylation of cellular proteins rather than the inhibition of cholesterol synthesis [173]. Geranylgeranylation is a post-transcriptional modification that covalently attaches geranylgeranyl to various cellular proteins to facilitate their membrane association. These geranylgeranyl groups are isoprenoids synthesized in the cholesterol biosynthesis pathway. More recently, FBL2 has been reported to be a host target for geranylgeranylation. Geranylgeranylation of FBL2 appears to be critical for HCV replication because the association between FBL2 and NS5A, an interaction that is a prerequisite for HCV replication, depends on geranylgeranylation of FBL2 [174]. *In vivo* results of statin monotherapy have yielded conflicting results so far. Neither atorvastatin (after conventional 12-week therapy) nor rosuvastatin resulted in a reduction in viral load [175,176]. Nevertheless, fluvastatin was found to inhibit HCV RNA replication in HCV infected patients in a recently published study. The drug was well tolerated and resulted, at relatively low doses (20–80 mg daily), in a transient reduction in viral load (−0.5 to −1.75 log_10_, 2–5 weeks); higher doses of fluvastatin did not reduce viral load [177]. Also, a recent retrospective analysis demonstrated that statin use in combination with pegIFN and RBV was associated with a significantly higher SVR and viral response at week 4 and 12 [178]. A pilot study evaluating the combination of fluvastatin (20 mg daily) with pegIFN/RBV revealed that fluvastatin could be used safely to increase the response to pegIFN and RBV [179]. There is today obviously no compelling evidence that statins, used in monotherapy, may result in a marked reduction in HCV load in chronically infected patients. However, statins may have the potential to increase the efficacy of current or future HCV therapy.

#### 2.2.4. Nitazoxanide (Alinia®, Romark laboratories)

Nitazoxanide (NTZ) is a thiazolide with activity against bacteria, protozoa and viruses. The antiviral activity of NTZ against HCV was discovered during its development for the treatment of cryptosporidiosis in patients with acquired immune deficiency syndrome (AIDS) coinfected with HCV [180]. NTZ and its primary metabolite, tizoxanide, inhibit HCV replication *in vitro* but do not induce antiviral resistance, since direct HCV viral resistance did not emerge with serial exposure to NTZ, suggesting that the genetic barrier of resistance to NTZ is high [181 ,182]. Although the mechanism of action is not known, it is assumed that the activity is related to (a) cellular rather than (a) viral target(s). NTZ has been reported to increase the phosphorylation of dsRNA-dependent protein kinase thereby resulting in the inducing of initiation factor 2 α which is an important factor in innate immune response.

The antiviral effect of NTZ in HCV-infected patients was first reported in 2008 [183]. In genotype 4 infected patients NTZ monotherapy (500 mg, BID) proved safe and resulted in a sustained virological response in 17.4% of patients. In another study, triple therapy with pegIFN, RBV and NTZ (180 μg pegIFN once weekly, 1000–1200 mg RBV/day, 500 mg NTZ, BID) for 36 weeks following a 12-week lead-in with NTZ monotherapy (500 mg, BID) resulted in a SVR of 79% in treatment-naïve patients infected with HCV genotype 4, which was superior to the SVR rate achieved with the SoC using pegIFN and RBV for 48 weeks (SVR=50%) [184]. Currently two clinical trials are being performed, e.g. STEALTH-C2 and C3, in which the combined effect of NTZ (500 mg, twice daily) with RBV and pegIFN is studied in genotype 1 infected prior non-responders to pegIFN/RBV therapy or in genotype 1 treatment-naïve patients, respectively. Interim results of the ERAIS-C trial revealed that a regimen of NTZ (500 mg, BID), high dose RBV and pegIFN enhances rapid viral response (RVR) [185]. Another pilot study of lead-in NTZ enrolled cirrhotic patients with chronic HCV genotype 1 who failed previous therapies with pegIFN and RBV. These patients received NTZ 500 mg twice daily for 4 weeks followed by NTZ plus pegIFN 180 μg/week and weight based RBV for 48 weeks. An interim analysis of this study indicates that treatment with lead-in NTZ in combination with pegIFN and RBV has demonstrated promising results in these difficult to treat patients [186].

#### 2.2.5. Silymarin Extracts

Silymarin is a mixture of flavolignans extracted from the milk thistle (*Silybum marianum*). The main component is silibinin (a 50:50 mixture of silibinin A and silibinin B). Silibinin has strong anti-oxidative and anti-fibrotic properties and has therefore been used for many years as a ‘hepatoprotector’ agent. Silibinin A and B, as well as Legalon SIL, a commercially available intravenous preparation of silibinin, exhibited an antiviral effect in the HCV replicon system [187]. Furthermore, these products, as well as a standardized silymarin extract (MK-001), inhibited infection of Huh 7 and Huh 7.5.1 cells by the JFH-1 HCV strain in a dose-dependent manner [188]. Recently silibin and related compounds were found to directly inhibit HCV NS5B polymerase [187]. *In vivo* results of silymarin extracts have yielded conflicting results so far. When Legalon SIL was administered intravenously for 7 days at 20 mg/kg/day, HCV RNA was reduced by 3.02 ± 1.01 log_10_ in non-responder patients [189]. HCV RNA decreased further after 7 days of combined treatment of SIL with SOC (4.85 ± 0.85 log_10_). Beside mild gastrointestinal symptoms, IV silibinin therapy was well tolerated. However, when the antiviral effect of oral silymarin extracts was studied, no antiviral effect was found in HCV infected patients [190,191]. The discrepancies between previously mentioned studies are probably due to the dose and route of administration of the drug. Recently it was shown that high-dose administration of oral silymarin was associated with steady-state peak plasma concentrations of silibinin A and B 1 to 2 orders of magnitude below the concentrations associated with antiviral effects *in vitro*. Moreover, trough concentrations were approximately 25-fold lower than peak concentrations, likely explaining the lack of antiviral effect of oral silymarin *in vivo* [191].

## 3. Combination Therapy

As HCV has a high replication rate without a proof-reading mechanism, the emergence of viral resistance forms a major issue within this class of specifically HCV-targeting molecules. To stand up to this problem combinations of molecules with different modes of action will play a key role in the future development of these compounds.

Recently, various studies were performed to investigate the antiviral efficacy of combinations of HCV inhibitors *in vitro*. Selection of resistance of the combination of R1479 or PSI-6130 with a protease inhibitor or non-nucleoside inhibitor reduced the emergence of resistant colonies, suggesting a clear benefit of combination treatment [192]. When selecting replicons with dual resistance to inhibitors binding to the thumb and palm sites, combination experiments showed that dual-resistant replicons had amino acid substitutions that mapped directly to both the thumb and palm sites. Thus, combination therapy apparently did not significantly complicate the resistance profiles observed with single-compound treatments [114]. Combinations of inhibitors with a viral and a cellular target were also studied *in vitro*. The combined *in vitro* antiviral activity of Debio 025 and RBV or selective HCV inhibitors was shown to be additive and Debio 025 prevented resistance development against several STAT-C inhibitors [193]. Another HCV inhibitor with cellular target, mevastatin, delayed or even prevented the development of HCV-796 escape mutants [172].

The INFORM-1 trial is the first trial to study the combination of a nucleoside polymerase inhibitor (R7128) and a protease inhibitor (ITNM-191/R7227) in HCV infected patients. Both compounds inhibit different targets and are therefore good candidates for combination therapy. HCV genotype 1 infected patients received up to 14 days oral combination therapy with escalating doses of either 500 or 1000 mg BID R7128 & 100, 200 mg TID or 600 or 900 mg BID R7227, leading to a median HCV RNA change from baseline between −3.5 and −5.2 log_10_ IU/ml at day 14 [194]. Low dose combination therapy of R7128 with R7227 did not select for resistant mutants for up to two weeks of treatment [195].

## 4. Discussion

Important progress in the development of potent and selective inhibitors of HCV replication has been made in the last decade. Several (relatively) well tolerated drugs are currently in clinical development and encouraging results have been obtained with some of these molecules. The ultimate goal is to develop a successful therapeutic strategy against most, if not all, HCV genotypes that results in a rapid and potent antiviral response and that does not, or only to a limited extent, allows the virus to develop drug-resistance. Such therapy would no longer require the use of ribavirin and interferon preparations, therapeutics that are associated with important side-effects. Drugs to be used in combination therapy must have non-overlapping resistance profiles. This is achievable, given the fact that inhibitors have been/are being developed that target (i) different steps in the viral life cycle (amongst which entry, the NS3 protease, the NS5B polymerase and NS5A) and (ii) cellular factors that are crucial for efficient viral replication (e.g., the non-immunosuppressive cyclophylin binding molecules). So far, inhibitors of the viral NS3 protease and NS5B polymerase as well as the viral NS5A (of which the precise function in the replication of the virus still remains to be unraveled) have been best studied and their value has been demonstrated in the clinical setting. Most polymerase inhibitors as well as the NS5A inhibitors have been discovered by (i) screening compound libraries in either target based assays (eg. RdRp assays) or in cell based assays (eg subgenomic replicons) followed by (ii) hit-to-lead optimization. NS3 protease inhibitors have basically been designed as peptidomimetics of the carboxyterminal domain of the cleavage site of the enzyme. It is somehow surprising that very few inhibitors of yet other targets, such as the NS4B, the NS3 helicase activity or the interaction between NS4A and the NS3 protease have not been identified. The reason, in particular for the viral helicase, may be that the molecules that are usually present in small molecule libraries have not the right scaffold (or may be too small) to exert activity against these targets.

The viral polymerase has multiple targets for inhibition of viral replication. Besides the catalytic site, different allosteric sites exist that can each be targeted by specific inhibitors. In theory, a combination of two or three different NS5B RdRp inhibitors may thus suffice to design a combination therapy. In fact, Atripla, a HAART (highly active anti-retroviral therapy) consists of three inhibitors of the HIV reverse transcriptase, each with a different resistance profile. *In vitro* studies may help to develop combinations that are highly potent against HCV. Such combinations should also delay or prevent the development of resistant variants. Pharmaceutical companies that have two or more compounds in (clinical) development with non-overlapping resistance profiles may have the option to develop such combination therapies. Strategic partnerships of different companies that each owns one (or two) compounds that would be ideally combined with yet another inhibitor may possibly be needed to obtain the most powerful combination therapy.

## Figures and Tables

**Figure 1.**
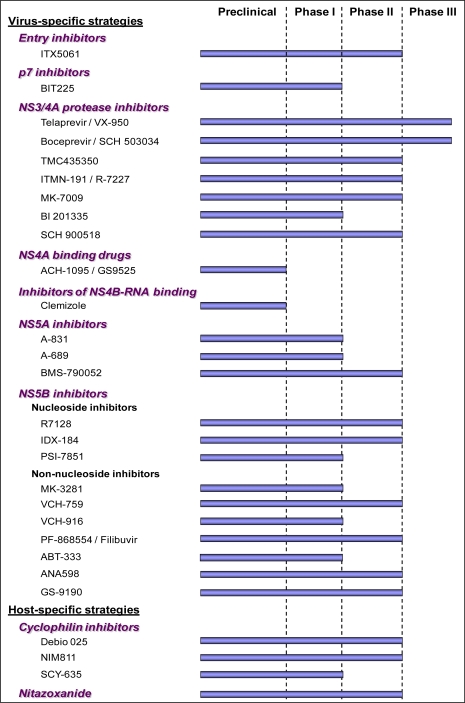
New antivirals for the treatment of HCV and current stage of development.

**Figure 2.**
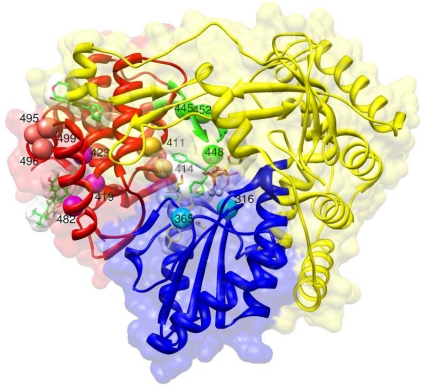
Binding sites for HCV polymerase inhibitors. This figure is created using Chimera [105] based on superposition of different pdb structures using the Dali server [106]. The superimposed structures are 1GX6 (active site with UTP), 2BRK (with benzimidazole in thumb domain site 1), 1OS5 (with dihydropyranone in thumb domain 2 site), 1YVF (with benzothiadiazine in palm domain site 1), 3FQL (with benzofuran in the palm domain site 2). The palm, fingers and thumb domains are colored blue, yellow and red respectively. UTP and benzofuran have yellow carbons, the other molecules green carbons. Mn++ ions in the active site are grey. Mutations in different regions have a different color: salmon for residues P495, P496, V499 in thumb domain 1, magenta for L419, M423, I482 in thumb domain 2, gold for N411, M414 in palm domain 1, cyan for C316, S365 in palm domain 2, green for C445, Y448, Y452 in the beta-hairpin loop.
